# Learning to Represent a Multi-Context Environment: More than Detecting Changes

**DOI:** 10.3389/fpsyg.2012.00228

**Published:** 2012-07-20

**Authors:** Ting Qian, T. Florian Jaeger, Richard N. Aslin

**Affiliations:** ^1^Department of Brain and Cognitive Sciences, University of RochesterRochester, NY, USA; ^2^Department of Computer Science, University of RochesterRochester, NY, USA

**Keywords:** multi-context environment, contextual ambiguity, representation learning, contextual cue, change detection

## Abstract

Learning an accurate representation of the environment is a difficult task for both animals and humans, because the causal structures of the environment are unobservable and must be inferred from the observable input. In this article, we argue that this difficulty is further increased by the multi-context nature of realistic learning environments. When the environment undergoes a change in context without explicit cueing, the learner must detect the change and employ a new causal model to predict upcoming observations correctly. We discuss the problems and strategies that a rational learner might adopt and existing findings that support such strategies. We advocate hierarchical models as an optimal structure for retaining causal models learned in past contexts, thereby avoiding relearning familiar contexts in the future.

## 1 Introduction

Learning requires a mechanism that infers from observable events in the environment a minimally sufficient hypothesis of the unobservable underlying structures. This hypothesis not only serves as an efficient representation of the causal relations in the environment, at least for a particular task, but also enables the learner to generalize to events that have not been observed. For example, if the task involves predicting the consumption of different food items in a school cafeteria, then a reasonable approximation is to tally the quantity of each food item that was consumed over some running average of the past (e.g., the prior month). However, there is considerable variation in these tallies across hours of the day, days of the week, and specific occasions such as holidays. Thus, in order to prevent more than the occasional dissatisfied customer, the manager of the cafeteria must develop a fairly flexible model that can modulate its predictions of the demand for food items dynamically given the values of these key variables. We will refer to these key variables as *contexts* and the cafeteria environment as an example of a *multi-context* environment. Each context in such an environment is associated with a distinctive causal structure. In the present article, we argue that most realistic environments are inherently multi-context, and that learning a flexible model that embeds information about contexts is the general task that confronts naïve learners. To successfully accomplish this task, learners must be able to (1) infer (with uncertainty) whether a context change has occurred; (2) adapt to a changed context and learn new causal models if necessary; and (3) represent contexts along with corresponding causal models in an optimal manner.

Context changes often signal that a different underlying causal model now applies. However, contexts are rarely explicitly labeled in the input available to the learner, and many contextual cues that are easily observable are not relevant to the underlying causal model. The canonical case, then, involves implicit contexts that must be discerned by the learner, often by noting that the current causal model does not provide an adequate fit with the most recent input. Thus, the first challenge of learning in a multi-context environment is to detect context changes from unexpected observations alone. This would be a trivial problem if the causal relations within each context were strictly deterministic. Consider the cafeteria example again. If the consumption rate of bottled milk during breakfast hours is exactly 10 bottles per minute, it is not difficult to conclude that breakfast is over when the rate drops to 1 bottle per minute. However, such deterministic relations are rare in reality. It is possible that the *average* consumption rate of bottled milk is 10 bottles per minute during the breakfast context, but occasionally, it might be as low as 2 bottles or as high as 20. The uncertainty resulting from random and probabilistic variations creates a difficult situation for the manager: if a large lecture class, originally scheduled at 9 A.M., is canceled because the professor’s return flight from a conference is delayed by bad weather, then the demand for milk at the cafeteria may be altered idiosyncratically – the manager may observe a decrease as students are likely to get up later and skip breakfast. Unaware of the implicit context (i.e., class canceled), the manager is now faced with the problem of *contextual ambiguity*: should the manager interpret this decrease as acceptable random variations in the regular breakfast context or as the representative characteristic of a changed context?

Resolving contextual ambiguity is only the first step of learning in a multi-context environment. Once a learner arrives at the conclusion that a different context has come into effect, they must also decide how to adapt to the changed context. Here, a learner has at least two choices. They can either learn a new model and associate it with the context, or retrieve from memory a causal model learned for a past context, which closely resembles or even matches the current context. The need to learn a new causal model arises when the learner encounters a novel context. Consider a *new* manager of a school cafeteria. Although the new manager may draw upon her experience of working in a cafeteria at a different university, there remains the possibility of encountering novel contexts on the current campus. For example, students at the current university may prefer sleeping in over attending classes on Friday mornings, which would require reduced stocking of bottled milk on those days. Like a naïve learner in any task, the new manager not only has to learn the average quantity of milk to stock (i.e., the model), but also has to associate it with Friday mornings (i.e., the appropriate context). The difficulty lies in the fact that there are often no explicit cues for the manager to gain sudden insight into *what the appropriate context is*: Instead of using friday morning, the manager could just as easily consider the weather on that particular day. The benefits of identifying the appropriate contexts, on the other hand, also extend to the second choice of adapting to the change in context: reusing a learned model. If the learner has correctly associated the causal model (e.g., decreased demand for bottled milk) with the relevant context (e.g., friday morning), then, in theory, they will be able to retrieve and reinstate the model when the target context is effective again (e.g., next Friday).

Assuming that the learner has the ability to reinstate a previously learned causal model, does it mean that the learner must be capable of storing and representing multiple contexts simultaneously? Although intuitively, the answer to this question has to be a strong “yes” (since learning a new causal model should not lead to elimination of an old one), it is not immediately transparent how these multiple contexts and their corresponding causal models are organized in the mind of the learner. Are contexts represented without order, as in “a bag of contexts/models,” or are they structurally organized? For example, do learners represent the relations between different contexts so that the changes in one context may be generalized to another? A rational approach might predict that contexts with similar causal models are clustered to achieve an efficient representation as well as to highlight the relationships among contexts. How can these intuitions be captured in a formal model for learning in multi-context environments?

In the rest of this article, we integrate existing findings that are relevant to the issue of learning in a multi-context environment. Our primary goal is to offer a comprehensive overview that brings together insights from across various literatures of cognitive science, so that one may come to realize what is yet to be investigated and understood. To avoid potential confusions, we distinguish the multi-context learning environment we are interested in from the partially observable Markov decision process (POMDP) that often concerns the reinforcement learning community (Stankiewicz et al., 2006; Gureckis and Love, 2009; Knox et al., 2011). In a POMDP problem, the environment implicitly transits from one state to another as a function of its past states and subjects’ actions. The learner must infer the current state they are in and how states change in order to take appropriate actions and maximize gains. Despite the apparent similarity between “state” and our notion of a “context,” a POMDP by itself is not a multi-context environment. This is because once the learner has successfully discovered the representation of the underlying Markov process, they will have an optimal, and most importantly, stable solution for maximizing gains over time, as long as the underlying Markov process does not change. Our discussion here, as illustrated by the cafeteria example, focuses on exactly the opposite case: that the underlying process, be it a Markov process or a simple generative model without temporal dependencies, changes unpredictably over time, rendering any previously learned model insufficient for the new context.

Additionally, we outline the directions for future research. How the learner determines when a change in context is relevant and then learns a new causal theory must, we claim, involve building hierarchical models (or heuristic approximations of them). Such a hierarchical model must include the storage of multiple contexts so that the unexpected input serves as a trigger to shift from one causal model to another, rather than simply updating the current model to improve the fit. Finally, we hypothesize that contexts themselves are structurally rich components that may share *cues*, so that it is possible to infer whether the environment has returned to a previous context at the time of a context change.

## 2 Detecting a Context Change

In a realistic learning task, the learner has to rely on observations that unfold over time to form hypotheses about the environment. If the environment consists of a single-context, the sequential nature of the input is less likely to be a problem since an optimal learning strategy, as prescribed by Bayesian belief updating, is available (for general discussions on Bayesian modeling of cognition, see Griffiths et al., 2008; Jones and Love, 2011). Similarly, if the learner is given explicit information regarding which context they are currently in, there are no contextual ambiguities to solve. However, in most cases (such as the cafeteria example), the environment might change from one context to another implicitly, leaving the learner with the difficult task of estimating where one context ends and another one begins. The difficulty is further compounded by the sequential availability of the input – recognizing the emergence of a different context must be achieved in an on-line manner rather than with *post hoc* analysis. Detecting context changes is commonly referred to as a *change detection* problem in many studies (e.g., Behrens et al., 2007; Yu, 2007).

While monitoring for unexpected observations in the input is an intuitive strategy for detecting context changes, at the core is the problem of interpreting ambiguity in the unexpected data: they can be interpreted as outliers if we assume the environment is still in the same context as before, or, they can also be interpreted as representative samples of a new context that is already in effect. As mentioned in the Introduction, we refer to this type of ambiguity as *contextual ambiguity*. How do learners resolve contextual ambiguity? Can they do so optimally? A satisfying answer to these questions requires a definition of optimality in the context of resolving contextual ambiguity. We discuss the factors that have been shown to influence how the learner resolves contextual ambiguity before presenting our definition of optimal ambiguity resolution.

### 2.1 Prediction error

Prediction error is widely recognized as one factor that can be used to adjudicate between outliers versus a true context change. In typical experimental settings, prediction error is either explicitly signaled by the degree of reduction in reward on a trial-by-trial basis (i.e., the utility of an action; Behrens et al., 2007; Pearson et al., 2009; Nassar et al., 2010) or assumed to be (subconsciously) computed by learners who seek to optimize overall task performance (in which case the utility of the action is not explicitly known; e.g., Fine et al., 2010, submitted). Large prediction errors, especially when they persist over time, often imply a change in context, while small prediction errors are likely to be random deviations in the current context. Thus, on average, learners will resolve contextual ambiguity faster when the new context differs greatly from the previous context. In the animal conditioning literature, the partial reinforcement extinction effect describes exactly that situation – after the extinction of reward, animals stop displaying the conditioned behavior more quickly when the behavior was trained with a high reward rate than with a low reward rate (Tarpy, 1982; Pearce et al., 1997). Going from a high reward rate environment to the extinction stage results in larger prediction errors than going from a low reward rate environment. Similarly, during foraging, animals tend to stop visiting a depleted food source more quickly if the source location was previously associated with a high return of food (Kacelnik et al., 1987; Dall et al., 1999).

When human learners are tested in a similar experimental paradigm known as the “bandit game,” which features sequential choices among several alternatives with various reward rates, they tend to show higher learning rates when experimenters change reward rates without announcing the changes (Behrens et al., 2007; for similar results obtained from another experimental paradigm, see Nassar et al., 2010). Intuitively, high learning rates can accelerate the process of learning a new causal model, which helps quickly minimize the ongoing prediction error. The more important finding is, however, that the learning rate positively correlates with the magnitude of prediction error, where prediction error is measured in terms of either the utilities of actions (such as the difference between expected reinforcement and the reinforcement actually received; e.g., Courville et al., 2006) or the accuracy of directly predicting variables of interest (e.g., Nassar et al., 2010). This implies that human learners potentially react to context changes in an optimal (or at least near-optimal) fashion: with small prediction errors, the learner adjusts their current behavior conservatively since small errors are likely to be random variations; with large prediction errors, the learner adopts a high learning rate to catch up with what is probably a changed context. Such behaviors can be qualitatively predicted by rational models that anneal learning rates based on the magnitudes of prediction errors, such as the Kalman filter. In experiments where the normality assumption of the Kalman filter does not apply (Yu and Cohen, 2008) have successfully applied the linear-exponential filter to describe subjects’ behaviors in a multi-context categorical learning task.

Converging evidence for the role of prediction error is also provided by imaging and multi-electrode recording studies. It has been suggested that the brain region known as the anterior cingulate cortex (ACC) represents prediction errors at the time of outcome (see Yu, 2007; Rushworth and Behrens, 2008, for reviews and opinions on the role of ACC) or related quantities (e.g., the “volatility” of an environment; Behrens et al., 2007). More recent studies also suggested that the neurons in the ACC may be more accurately described as tracking the surprisal of an event rather than the magnitudes of reward prediction errors *per se* (e.g., Hayden et al., 2011). In other words, the ACC seems to be involved in accurately predicting upcoming events, rather than reacting to changes in the utilities of actions in the environment.

In the above scenarios, the information about prediction error is assumed to be immediately available once the learner has made a decision. However, there are other cases where such an assumption does not hold. For example, when prediction errors are derived from rewards, the learner will experience delayed prediction errors if rewards are given out in batches rather than on a trial-by-trial basis. How should the learner detect a context change in these situations? If learners adopt the same strategy as in an environment with immediate feedback, the overall loss will likely be widened because the incorrect causal model will be applied for a much longer period of time. So far, little empirical research has been conducted to investigate what kinds of strategies learners actually use to detect context changes in an environment coupled with delayed prediction errors.

### 2.2 Estimation uncertainty

Although large and small prediction errors are correlated with different presumed explanations for outliers, there are two types of prediction errors that are worth distinguishing. In the first case, the learner makes a substantial number of prediction errors because a good model of the environment has not yet been formed. Those prediction errors are the result of random guessing and are thus unhelpful for the purpose of resolving contextual ambiguity. The other type of prediction error arises when the learner is confident that the current causal model has been sufficiently refined to be a good theory for the current context, and then becomes genuinely surprised by the inadequate fit with the most recent input. From the rational decision-making perspective, only this second type of prediction error is meaningful to the learner (the solution to the former is simply to collect more data). However, its effect might seem counter-intuitive to those who are familiar with the Kalman filter. In the Kalman filter, the influence of a large prediction error will be lessened if the observer is confident about current estimates. Yet, this balance between prediction error and estimation uncertainty is only rational if the environment is assumed to be stationary. When there is more than one context in the environment, large prediction errors at the time of low estimation uncertainty should indicate the emergence of new contexts. To test this hypothesis, one expects that when facing a particularly difficult task (due to either complexity or limited sampling), learners will be less likely to reach a low-uncertainty estimate of the current causal model, and they will consequently fail to recognize new contexts as easily as they have done in the studies reviewed above.

Unfortunately, none of the studies that we are aware of have addressed this issue directly within a single experimental paradigm. However, an artificial language learning experiment has provided some interesting insights. In Gebhart et al. (2009), learners listen to two artificial languages presented successively in a single session (with equal amount of exposure and without an overtly signaled change point). Under these conditions, only the first language is learned. The crucial difference between artificial grammar learning paradigms and simple decision-making tasks (such as the bandit games in Behrens et al., 2007) is that learners in the latter environment are able to reach asymptotic performance relatively effortlessly. On the contrary, learners cannot easily reach asymptotic performance in an artificial grammar learning experiment due to the high-dimensional nature of the linguistic input (Gerken, 2010). Therefore, the high uncertainty associated with the model of the first language prevents the learners from resolving the contextual ambiguity and learning a second grammar. Another experiment, in which subjects were tested with a variant of the famous Wisconsin Card Sorting task, showed that learners failed to detect when the sorting game entered a new context (characterized by changes in the reward rules) as optimally as a Bayesian learner (Wilson and Niv, 2012). Presumably, this is also because it is difficult to reach low estimation uncertainty when context changes result in structural differences in the causal relations, which is a more demanding learning task. Future studies, however, must test the hypothesis of estimation uncertainty directly within a single experimental paradigm to further our understanding of this issue.

### 2.3 Prior expectation for context change

What happens if learners approach the problem of resolving contextual ambiguity with a bias toward looking for changes in context? Put differently, will believing that there are multiple contexts prior to learning improve the recognition of changes? A variant of the foregoing artificial language learning experiment was conducted, where not only the subjects knew that there would be two languages (i.e., contexts), but also they experienced a 30-s silent pause between these two languages (Gebhart et al., 2009). With this change, subjects readily learned both languages. The bias toward changes can also be introduced by the use of more subtle explicit cues (e.g., subjects learn separate models when each context is coupled with a speaker-voice cue: Weiss et al., 2009), or by familiarizing learners with the pattern of a multi-context environment prior to conducting the target trials (Gallistel et al., 2001). These findings suggest that the prior expectation for a change in context enhances the ability of recognizing context changes in subsequent sequential input.

Is having a prior expectation for changes in context beneficial for learning in realistic and ecologically valid environments? This is largely an empirical question that awaits further experimental investigation (see Green et al., 2010 for relevant discussions). Theoretically, it is not difficult to see that such a prior expectation is only advantageous when it matches the frequency of context changes in the environment. If the prior expectation for context change is comparatively weak, learners would simply ignore contextual ambiguity and miss the new context. However, if it is too strong, learners may effectively treat each minor deviation as a signal for a new context in the environment – thus over fitting the data. In that case, no stable learning can be achieved.

The ideal solution for the learner would be to estimate the frequency of context changes in the environment before learning begins. However, such a strategy is only possible when the learner is familiar with the task environment and can anticipate the start of the learning process. Estimating the frequency of context changes in a *novel* environment, whose cues and features are entirely different from what the learner has encountered before, is indeterminate because there is no certainty about the type of changes and when they occur. The question of interest is then: how strong a prior the learner has for context changes in these novel environments? While experimental evidence on this issue is thin, we do know that prior expectations for context change, in the absence of explicit instruction from the experimenter or explicit cues from the environment, must be relatively moderate. Such insights come from experiments where the context of the environment alternates frequently, resulting in an unrealistically volatile causal structure. In those conditions, learning is either virtually non-existent (Clapper and Bower, 2002) or substituted by a heuristic strategy that heavily depends on recent exemplars (Summerfield et al., 2011). The tendency of preferring locally stable and coherent observations is also seen in young infants: in the absence of suggestive information, infants are more likely to assume that a sequence of observations consists of correlated samples with common properties rather than independent samples randomly drawn from the whole population (Gweon et al., 2010).

## 3 Adapting to the Changed Context

Once a context change is hypothesized to have occurred, the learner must decide how to adapt to the changed context. If the context is novel, the learner has no choices other than to infer a set of new causal relations from observations. If the context is familiar, however, the learner may retrieve from memory the causal model of a past context and use it to predict future observations (c.f. Freidin and Kacelnik, 2011). Instead of discussing both scenarios directly (which we will cover slightly later), here we focus on two theoretical assumptions that must be in place to make these scenarios possible: the capacity of storing multiple contexts and the organization of these contexts in memory.

### 3.1 In with the new, while retaining the old?

When the environment presents a novel context, a new causal model should be generated to represent the dependencies between the variables of interest. To achieve this goal, the learner can either update the current causal model, parametrically or structurally, or learn a second model that will co-exist in parallel with the previous one. Existing accounts, such as associative strength theories (e.g., the Rescorla–Wagner model; Rescorla and Wagner, 1972) or reinforcement learning models (see Payzan-LeNestour and Bossaerts, 2011 for an example), have typically assumed the former theoretical position. Such a theoretical position is also shared by the more recently proposed change detection models (see Box 1) and sequential sampling models (see Box 2), both of which are intended to explain how ideal learners should behave in multi-context tasks.

**Bayesian change detection models.**

Detecting a change in context is an important step in learning a rich representation of a multi-context environment. The traditional approach to change detection comes from studies of controlled stochastic processes (e.g., Shiryaev, 1978), where the goal is to find an optimal policy for mapping observations to stopping decisions (i.e., whether or not to consider that a context has ended). While the solutions are useful for many engineering applications, it is often difficult to attach a cognitive interpretation to the algorithms used in those solutions.

Here we focus on the Bayesian change detection approach that has recently become popular in the cognitive science community. As a computational-level theory, these models describe how a rational observer *should* learn a causal model given a particular formulation of the problem (Marr, 1982). Consider a simple scenario where the goal is to predict the number of automobiles that pass through a given intersection in each 24-h period. The parameter of interest is θ, which refers to the number of automobiles being driven from point A to point B. The causal model to be discovered by the learner specifies the relation between the parameter θ and the observation *y*, the number of automobiles passing through the intersection. However, at any given time step, a change in context might happen (e.g., road construction), which will alter the previous relation in effect and yield unexpected observations. Detecting the change then depends on how likely the learner is to attribute the unexpected observations to a change in the value of θ. The change detection approach assumes the determining factor here is the learner’s expectation of the volatility of θ. If θ is assumed to be changing smoothly and with little variance (i.e., non-volatile), then learners will tend to view unexpected observations as outliers and keep the value of θ unchanged. If θ is assumed to be capable of abrupt changes of substantial magnitude, learners will more likely update the value of θ when observing unexpected data.

Formally, the volatility of an environment, represented by a hyper-parameter α, can range from 0 to 1: With probability α, θ*_t_* will be the same value as θ_*t*−1_; with probability 1 − α, θ*_t_* will be randomly drawn from a predefined reset distribution *p*_0_. Thus, if α is 1, then learners are essentially assuming a single-context environment, where the value of θ is the same at each time step. If its value is 0, then learners are essentially assuming a completely chaotic multi-context environment, where the value of θ at the preceding time step has no predictive value over the current time step at all. Any intermediate value reflects the degree to which learners are biased against single-context environments. Additionally, the value of α, i.e., the degree of volatility, can change over time as well.

This model gained its popularity due to its conceptual simplicity and the range of phenomena it can explain (Cho et al., 2002; Yu and Cohen, 2008; Wilder et al., 2010; Wilson et al., 2010; see also Nassar et al., 2010; Mathys et al., 2011) for variants that are claimed to be cognitively more plausible; and (Summerfield et al., 2011; Wilson and Niv, 2012) for cases where the Bayesian change detection model is not the best descriptor of human behavior). A significant drawback of this class of models, however, lies in its memory-less learning mechanism. Once the ideal learner detects a change in context, it learns the new parameter settings by overriding those of the old context. This is undesirable since animal and human learners have clearly demonstrated the ability of holding onto knowledge learned from past contexts.

**Sequential sampling methods.**

Sequential sampling models are another approach to learning in multi-context environments. These models are inspired by sequential Monte Carlo sampling techniques, which are commonly used to approximate Bayesian inference in analytically non-tractable problems. In the cognitive science community, the particle filter, one of the most common sequential sampling algorithms (e.g., Sanborn et al., 2010), has been successfully applied to learning tasks where there are changes in context (Brown and Steyvers, 2009). In a particle filter model, the learner is assumed to simultaneously entertain a limited number of hypotheses (called particles) about the values of parameters in the environment (in the limit, with an increasing number of particles, the filter approaches optimal Bayesian decision-making). This contrasts with the Bayesian change detection approach, where learners are assumed to maintain full uncertainty about the estimates of the volatility (i.e., α in Box 1) and state (i.e., θ in Box 1) parameters. Thus, the particle filter has been argued to approximate rationality in the literature (Sanborn et al., 2010). At the beginning of the learning process, random values of θ are assigned to the particles since the learner has not made any observation of the environment. Each particle is then repeatedly updated according to subsequent observations. If a particle reflects a theory of the environment that is consistent with a new observation, then it is likely to be retained. Otherwise, the particle is likely to be reset and its value resampled from the hypothesis space. Since this sampling process is stochastic, there is always some chance that a few particles are inconsistent with the current state of the environment. These inconsistent particles are useful for detecting context changes in the environment. When the learner encounters an unexpected observation, particles that used to be consistent with the previous context now need to be reset, while those that were previously inconsistent are retained and duplicated, thus achieving the goal of detecting changes.

While we are not aware of any study directly testing the different predictions made by the change detection and the particle filter models, one crucial difference exists between them. The particle filter model, due to its stochastic nature and its sensitivity to the order of sequential observations, is suited for predicting individual-level results (Brown and Steyvers, 2009; Yi and Steyvers, 2009; Frankenhuis and Panchanathan, 2011). The change detection model, because its goal is to characterize rational behaviors, is suited for predicting average behavior. Patterns of individual learning outcomes tend to be different from group-averaged learning outcomes (Newell et al., 2001; Gallistel et al., 2004). Particle filter models can readily accommodate such differences – a single run of a sequential sampler tends to yield unpredictable patterns, but the average of many runs, by definition, reflects the expected properties of the probability distribution that is being sampled from (see Daw and Courville, 2008, for a similar argument).

However, disrupting or erasing the causal model learned under a past context (also known as catastrophic interference in connectionist terms; French, 1999) might not be a rational choice, especially when the environment may revert back to a past context. Experimental findings suggest that animals and humans do not simply abandon knowledge of past contexts. For example, in conditioning experiments, animals that have gone through extinction still possess a trace of the learned dependencies between the conditioned stimulus and response, which can spontaneously recover (e.g., Sissons and Miller, 2009), be renewed (e.g., Bouton and King, 1983), or be reinstated (e.g., Thanellou and Green, 2011) under the right conditions. Adult barn owls can rapidly re-adapt to an abnormal association between auditory cues and locations in visual space if they have previously learned such abnormal audio-visual dependencies when they were young (Knudsen, 1998; Linkenhoker et al., 2005). Humans also routinely switch back and forth between a certain set of contexts, without relearning a causal model each time a previously encountered context is active (for example, becoming familiar with a foreign accent does not lead to a complete relearning of one’s native accent). It is impossible for learners to display such behaviors without, implicitly or explicitly, representing multiple contexts concurrently. In the domain of category learning, several connectionist networks, such as the ALCOVE model (Kruschke, 1992) and the SUSTAIN model (Love et al., 2004), and incremental Bayesian non-parametric models (Anderson, 1991) are both capable of representing multiple categories that are learned through sequential observations. Similarly, a theory that extends the representation of multiple categories to multiple contexts must also include a hypothesis about how these contexts are stored.

### 3.2 A bag of contexts?

Nevertheless, more behavioral and theoretical studies are needed to understand whether learners *optimally* represent learned models of past contexts, as would be predicted by a theory of a rational learner. When a past context has little to no chance of reappearing in the future, it seems unnecessary to store its information in memory (c.f. Anderson and Schooler, 1991). When a past context is quite common overall, or when a repetitive pattern of environmental changes has appeared, learners will benefit greatly if its information remains readily available through the learning process. In addition, in order to efficiently retrieve a causal model of a past context from memory, the learner must implement mechanisms that support the identification of familiar contexts. In the case where there are observable cues co-occurring with the advent of contexts, it is possible to index contexts with these cues for later retrieval. This is especially helpful as most contexts do not come with explicit labels – the use of co-occurring cues may serve as the functional labels for these contexts. As memory indices, contextual cues make the information learned in each context more easily retrievable (García-Gutiérrez and Rosas, 2003; Rosas and Callejas-Aguilera, 2006; Abad et al., 2009), and keep multiple contexts from interfering with one another (Lewandowsky and Kirsner, 2000; Yang and Lewandowsky, 2003). In the case where there are no cues whatsoever, we expect learners to have a more difficult time identifying familiar contexts, potentially because such identification would have to solely rely on assessing the fit of multiple existing models to observable data.

These types of optimal learning decisions call for a sophisticated theory that, in our opinion, must extend beyond a process of parameter or structural revision of a *single* causal model. This is because at the end of the day, the outcome of the learning process should be more than a snapshot of the latest context of the environment, but rather an organized body of knowledge summarizing various forms of causal relations in the environment, past, and present. We outline such a model – in the form of a Bayesian hierarchical model – in the next section. Finding the answers to these questions can greatly supplement our understanding of how animals and humans learn multiple causal models for multiple contexts to solve a particular task through sequential observations.

## 4 A Hierarchical Framework for Learning in Multi-Context Environments

The hierarchical Bayesian modeling framework has been successfully applied to a wide range of cognitive phenomena (e.g., Kemp et al., 2007; Kemp and Tenenbaum, 2008; also see Lee, 2011, for a review). In fact, most existing Bayesian models of change detection fall into the category of hierarchical models, where the volatility parameter is treated as a hyper-parameter (Behrens et al., 2007; and most notably the nested volatility model in Wilson et al., 2010). While we also advocate a hierarchical Bayesian approach for modeling learning behaviors in a multi-context environment, our primary goal is to understand whether the learner forms a hierarchical *representation* of the environment. Previous modeling efforts, on the other hand, have typically emphasized the issue of whether and how learners can dynamically adapt their strategies when contexts change. We argue that only when a generative model simultaneously represents multiple contexts and their corresponding causal models, will the ideal learner be able to attribute unexpected observations to the right sources, and retain and reuse causal models from past contexts (see Kording et al., 2007, for similar ideas).

**Figure 1:**
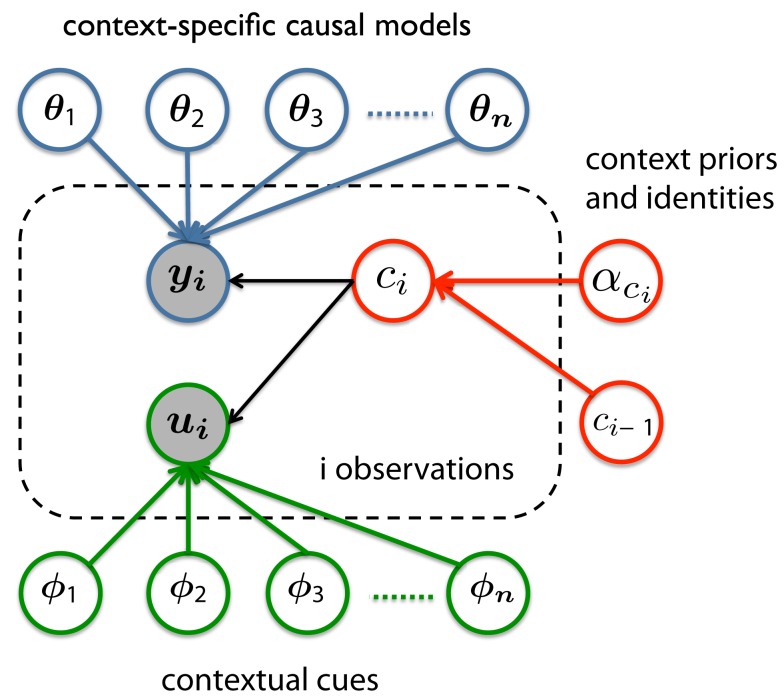
**One potential hierarchical model for representing information learned in a multi-context environment**.

Figure 1 shows one possible realization of such a hierarchical representation. For simplicity, consider an example where the causal models differ across contexts only in their parameter values, shown as **θ_1_, θ_2_, θ_3_, … θ_n_** in the figure (bold symbols denote vectors of variables). There are three components in this hierarchical representation. The first component (highlighted in blue) consists of the contexts and causal models, each of which describes a theory of how the observations of interest *y_i_* are generated from the parameters θ. Importantly, the parameters of the causal model of each context are individually represented, thus allowing for the storage of multiple contexts and avoiding catastrophic interference between these contexts. The second component is the mechanism that infers the identity of the currently active context *c_i_* (highlighted in red). This decision process in turn depends on two variables: the hyper-parameter $\alpha_{c_{i}}$ which reflects the likelihood of context *c_i_* coming into effect without explicit cues, and the inferred identity of the previously encountered context *c_i_−1*. The identity of the currently active context corresponds to only one of the causal models (i.e., one of **θ_1_, θ_2_, θ_3_, … θ_n_**). Thus, once the identity of the current context has been *correctly* inferred (which might not be true due to the probabilistic nature of the model), it can prevent the irrelevant contexts from being used to explain the observed data *y_i_* or being revised to fit unrelated data. In other words, the dependence between *y_i_* and *c_i_*, as shown in the figure, serves as a regulator that chooses the appropriate context as needed.

The third component in the hierarchical representation is the *optional* cuing mechanism (highlighted in green). When covarying cues *u_i_* are available, the values of these cues will depend on the identity of the contexts and the causal relations between contexts and these cues (the effect of Φ on *u_i_*). Therefore, these cues, in theory, serve the same functional purpose as the observations of interest *y* – evidence for inferring the identity of the current context. There is a vast literature on how humans may be able to optimally combine two sources of information to perform inferences (Ernst and Banks, 2002; Knill, 2007; Toscano and McMurray, 2010, to name a few). By building this cueing mechanism into the hierarchical representation, we are also making the assumption that learners should take advantage of the covarying cues as an extra source of information when available.

To be clear, Figure 1 is only meant to illustrate one of the many possible ways of constructing a hierarchical model to capture context-sensitive learning. Many details, such as the prior for the appropriate number of θ variables and any hyper-parameter reflecting the relationships between them, are not shown in the figure. Our goal here is to provide a concrete sense of what a hierarchical framework may look like for future modeling efforts. Experimental studies, especially those designed to test the effect of recognizing past contexts, are needed to further tease apart the factors that affect learning in a multi-context environment.

## 5 Considerations for Single-Context Laboratory Experiments

If animal and human subjects can readily detect new contexts without being explicitly instructed to do so, then we have reason to suspect that subjects will involuntarily look for context changes even in laboratory experiments where subjects are expected to learn a causal model for a fixed but unknown context. In a variety of such behavioral tasks, subjects exhibit an automatic and seemingly suboptimal behavior: they put an undue emphasis on the sequence of past observations, even when these observed stimuli are independent samples from the same causal model. Two notable instances of such suboptimal behavior in the literature are the hot hand illusion (Gilovich et al., 1985) and the tendency of reinforcing local patterns (e.g., Cho et al., 2002; Maloney et al., 2005; Gökaydin et al., 2011). While the conventional interpretation is that learners are irrational in that they perceive spurious correlations between past and upcoming outcomes, these seemingly suboptimal behaviors may well be the result of learners automatically inferring multiple contexts (e.g., hot hand context versus cold hand context) from the sequential input (for similar opinions, see Jones and Sieck, 2003; Yu and Cohen, 2008; Green et al., 2010; Wilder et al., 2010). More generally, the bias for perceiving multiple contexts may also hold the key to explaining order effects in learning (e.g., Sakamoto et al., 2008; Rottman and Keil, 2012). At the same time, it raises the concern that such a bias may lead to misinterpreted experimental findings because participants readily adapt to what they perceive to be changes in contexts (perhaps subconsciously). The above cited studies are in fact the best examples to show that the use of balanced designs in experiments do not effectively prevent participants from “inappropriately” adopting this bias (see Jaeger, 2010 for similar discussions).

## 6 Conclusion

Recognizing context changes in the environment helps learners build or choose the appropriate causal model and make accurate predictions about the consequences of their actions. In this article, we have addressed several questions about what we believe is the canonical case of learning: when the changes in context are implicit rather than being explicitly noted by a “teacher.” Current research findings suggest that learners are able to resolve contextual ambiguity and thereby recognize a new context by only observing sequential input, albeit with some limitations. Recognizing a new context is, however, only a part of the bigger picture. How do learners store the causal models of past contexts? Can learners reuse previously learned causal models? Crucially, given a change in context, should the learner build a new causal model or try to reuse, and potentially update, an old one? How should the learner decide? It is important to consider these questions when one attempts to define the expected behaviors of a rational naïve learner. We hope to address these intriguing questions in future research.

## Conflict of Interest Statement

The authors declare that the research was conducted in the absence of any commercial or financial relationships that could be construed as a potential conflict of interest.
